# Clear cell renal cell carcinoma metastasizing to the thyroid gland: a case report

**DOI:** 10.3389/fonc.2026.1752093

**Published:** 2026-04-13

**Authors:** Zhengshi Xia, Zuzhi Zhao, Peng Sun, Pengfei Xu, Jianhua Li

**Affiliations:** Department of Thyroid Surgery, The First Affiliated Hospital of Zhengzhou University, Zhengzhou, Henan, China

**Keywords:** case report, clear cell carcinoma, metastasis (cancer metastasis), renal cell carcinoma, thyroid metastasis

## Abstract

Metastatic tumors of the thyroid gland are rare in clinical practice. Clear cell renal cell carcinoma (ccRCC) is the most common primary site among metastatic thyroid carcinomas, accounting for 12% to 34% of all secondary thyroid tumors. Most secondary thyroid cancers have a poorer prognosis than primary papillary thyroid carcinoma. Therefore, the diagnosis and treatment of secondary thyroid tumors deserve attention. The diagnosis and treatment of secondary thyroid carcinoma pose significant challenges. We herein report a case of clear cell renal cell carcinoma metastasizing to the thyroid gland.

## Introduction

Thyroid carcinoma is a major global health burden. Most thyroid carcinomas are primary tumors, including Papillary Thyroid Carcinoma (PTC), Follicular Thyroid Carcinoma (FTC), Oncocytic Carcinoma (OCA), Differentiated High-Grade Thyroid Carcinoma (DHGTC), Poorly Differentiated Thyroid Carcinoma (PDTC), Anaplastic Carcinoma (ACA). Metastatic thyroid carcinoma is relatively rare in clinical settings. The kidney is the most common primary tumor site (33%), followed by the lung (16%), breast (16%), esophagus (9%), and uterus (7%) (1). The thyroid gland is an organ with a rich vascular structure. Theoretically, it should be a site where metastatic lesions are relatively easy to form, yet it is generally recognized clinically as an uncommon site for metastasis (2). Some scholars hypothesize that the rarity of metastatic thyroid tumors can be attributed to two factors. First, normal thyroid tissue has an abundant blood supply but lacks the ability to trap cancer cells, making it difficult for cancer cells to establish a foothold in this environment (3). Second, the relatively high levels of iodine and oxygen in the thyroid gland are not conducive to the proliferation of tumor cells, thereby exerting a certain degree of growth inhibition (4–6).

However, once a malignant tumor metastasizes to the thyroid gland, the prognosis is poor, the disease course is advanced, and it poses a great threat to life (7). Therefore, it deserves attention. Renal cell carcinoma (RCC) accounts for more than 50% of all clinically diagnosed metastatic thyroid carcinomas. Previous literature has reported multiple cases of renal cell carcinoma metastasizing to the thyroid gland (8).

Renal cell carcinoma (RCC) is the most common malignant tumor of the kidney. Many patients have already developed metastases when diagnosed (9, 10). Twenty-five percent of patients develop metastases after nephrectomy, and among patients with metastatic RCC, only 3% have solitary metastases (8).

## Case description

A 61-year-old male patient with a history of clear cell renal cell carcinoma was found to have a hypoechoic nodule in the right lobe of the thyroid gland during a local physical examination. Twelve years prior, he had undergone right nephrectomy for clear cell renal cell carcinoma. The specific staging is unknown. After the operation, the patient received three sessions of “biological therapy” and then had regular re-examinations for five years with no recurrence or metastases. A thyroid nodule found during a physical examination enlarged rapidly within one month. The patient then came to our hospital for treatment. The patient had no symptoms such as dysphagia, hoarseness, choking on drinking water, or local tenderness. Laboratory tests showed that all indicators were within the normal range except for a mild increase in free prostate-specific antigen (fPSA) and a significant increase in pepsinogen I and II (PG I/III). The specific values of thyroid function are as follows: Triiodothyronine (T3) is 1.46 nmol/L (reference range: 1.34-2.73 nmol/L); Thyroxine (T4) is 138.66 nmol/L (reference range: 78.38-157.4 nmol/L); Free Triiodothyronine (FT3) is 4.12 pmol/L (reference range: 3.28-6.47 pmol/L); Free Thyroxine (FT4) is 11.48 pmol/L (reference range: 7.9-18.4 pmol/L); Thyroid-Stimulating Hormone (TSH) is 1.478 uIU/mL (reference range: 0.56-5.91 uIU/mL). The specific value of Thyroglobulin (TG) is 24.10 ug/L (reference range: 3.5–77 ug/L). The patient had a history of hypertension for more than 20 years. Two years prior, he had undergone cardiac stent implantation at a local hospital. A follow-up ultrasound at a higher-level hospital revealed a cystic-solid nodule in the right lobe of the thyroid gland, measuring approximately 66 mm × 43 mm × 36 mm, classified as C-TIRADS Category 4a, with no obvious enlarged lymph nodes. The nodule has compressed the trachea. The indication for surgery was the size of the thyroid tumor. Due to the large size of the nodule and the absence of enlarged cervical lymph nodes shown by ultrasound, surgical treatment was required regardless of whether the nodule is malignant or not. The surgical resection scope included unilateral thyroid lobectomy plus isthmusectomy, as well as prophylactic central lymph node dissection (11, 12). Therefore, fine-needle aspiration (FNA) was deemed unnecessary. Preoperative routine computed tomography (CT) showed multiple small nodules in both lungs, of undetermined nature. During the operation, the parathyroid glands and recurrent laryngeal nerve were preserved. The patient had no special complications after surgery and was discharged on the 5th postoperative day. Before discharge, the patient was advised to go to the thoracic surgery department for management of pulmonary nodules and the necessary examinations. Twenty days later, the patient went to the thoracic surgery department for a PET-CT examination, which showed multiple solid nodules in both lungs. It considered to be metastatic lesions. The patient visited the thoracic surgery department a few days later as advised. The patient refused to undergo lung biopsy. After going to several hospitals, all the hospitals considered multiple pulmonary metastases. The thoracic surgery department did not recommend surgery. The patient was transferred to the oncology department for immunotherapy and targeted therapy. Up to now, the patient has received six cycles of treatment. The treatment plan was “Toripalimab 240mg intravenously infused on day 1, plus Axitinib 10mg orally twice daily, on a 21-day cycle”. Multiple pulmonary metastatic lesions have shrunk.

**Figure 1:**
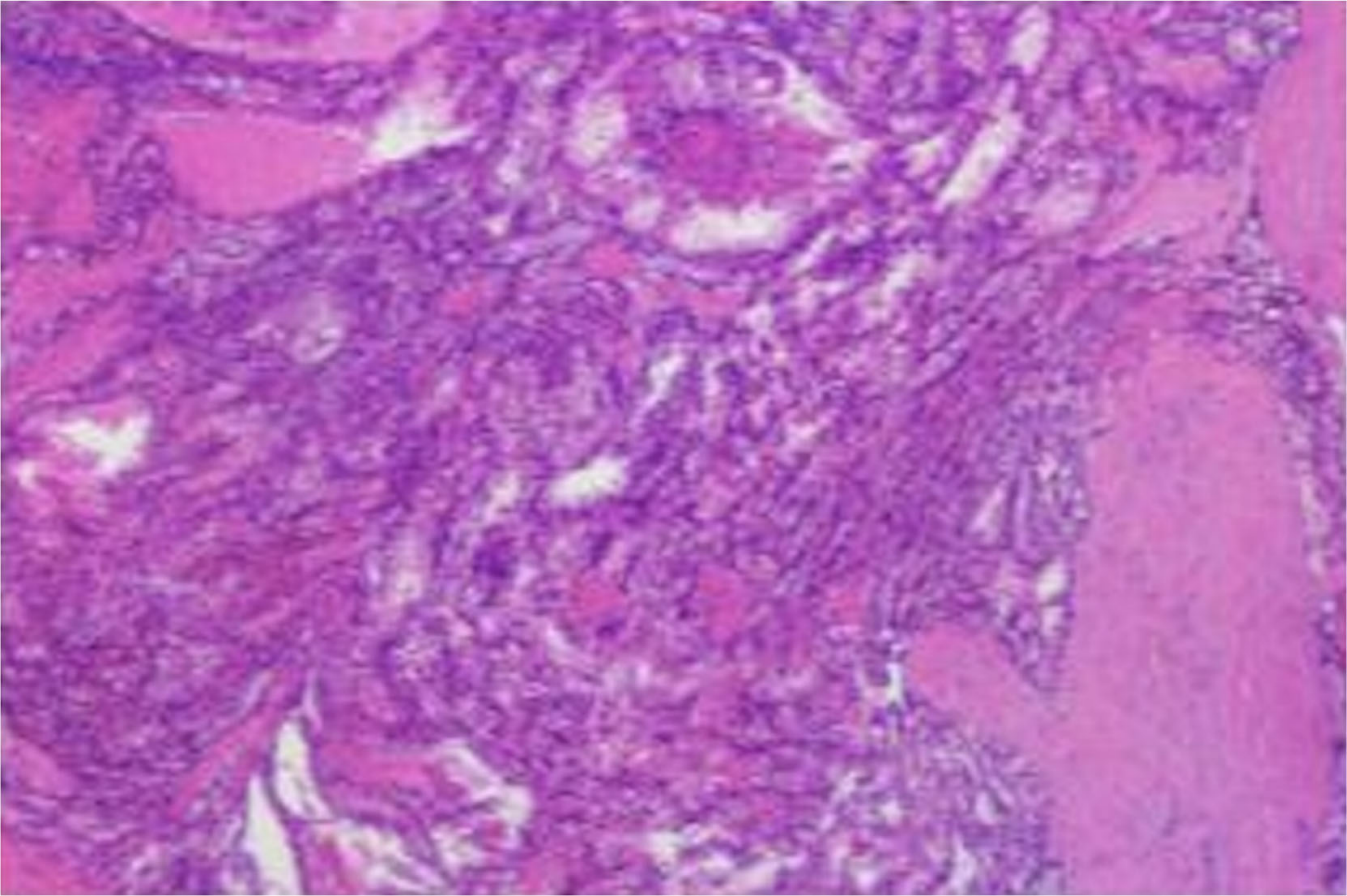
Microscopic findings of postoperative tumor tissue sections.

**Figure 2:**
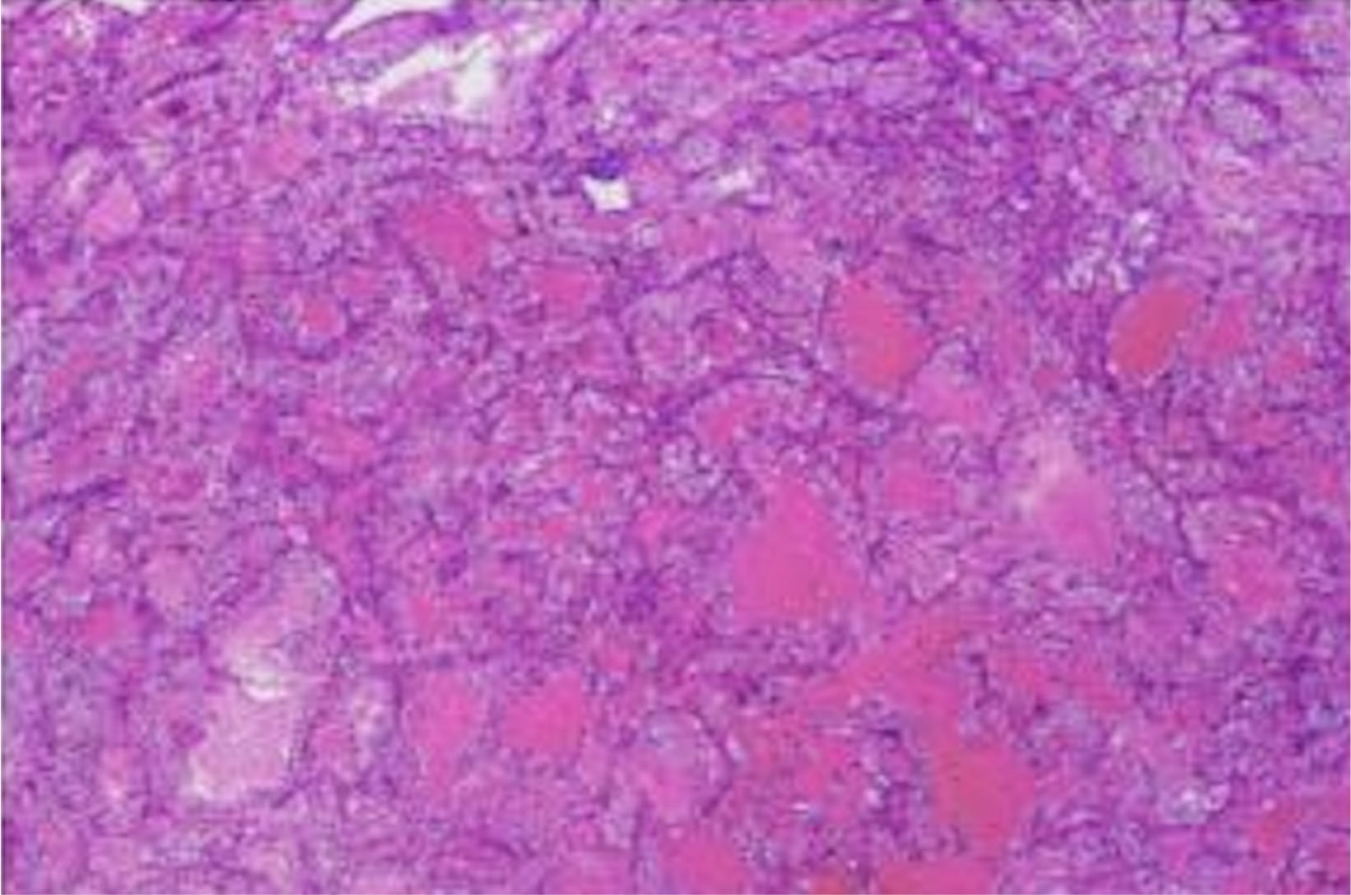
Microscopic findings of postoperative tumor tissue sections.

Postoperative histopathological examination revealed that there was metastatic carcinoma in the right thyroid gland, consistent with clear cell carcinoma metastasis based on morphology and immunohistochemistry (**Figures 1**, **2**). The immunohistochemical results showed the following: PAX-8 (+), TTF-1 (-), AE1/AE3 (+), EMA (+), Vimentin (+), CD10 (+), CA-9 (+), TFE-3 (-), Melan-A (-), HMB45 (-), CK7 (focal +), FH (+), SDHB (+), ALK (5A4) (-), and Ki-67 (approximately 5% +). Based on the postoperative pathological results, combined with the pathology of malignant tumors and relevant guidelines, it was recommended that the patient undergo additional surgical resection of the contralateral thyroid lobe. The patient refused to undergo reoperation (13–15).

## Discussion

Renal cell carcinoma (RCC) is a highly vascular tumor originating from the renal cortex, accounting for 90% of adult renal tumors. Many patients remain asymptomatic until the advanced stage of the disease, and approximately 25-30% of patients are diagnosed at an advanced stage or with multiple distant metastases. Common metastatic sites include the lungs (75%), regional lymph nodes (65%), liver (40%), and bones (40%) (8). However, RCC can also spread to unusual sites such as the thyroid gland, nasal cavity, larynx, and gums, making metastatic lesions in these sites easily overlooked (16). It should be noted that RCC may recur after many years (late recurrence).

For a newly detected thyroid tumor, it is crucial to distinguish whether it is primary or secondary. Secondary thyroid carcinoma, which is relatively rare clinically, results from the metastasis of distant malignant tumors to the thyroid gland. The primary lesions mostly originate from lung cancer, breast cancer, gastrointestinal tumors, RCC, etc. Most patients have a clear history of malignant tumors. The lesions are often multiple and bilateral. On ultrasound, they mostly appear as hypoechoic nodules, with relatively clear margins and rich blood supply, and generally do not have the typical features of primary thyroid cancer such as microcalcifications. The pathological morphology is consistent with that of the primary tumor. TTF-1 and TG are mostly negative, while they express markers specific to the primary tumor (17, 18). Identifying a primary carcinoma before surgery can help avoid unnecessary surgery. For patients with a history of primary carcinoma, attention should be paid to the possibility of metastatic carcinoma when diagnosing thyroid nodules. Both primary and secondary thyroid carcinomas can present with compressive or invasive symptom, such as dyspnea, dysphagia, choking on drinking water, and hoarseness, making such symptoms non-specific. Nodules show similar manifestations on ultrasound and radioactive iodine diagnosis, which also poses difficulties to diagnosis (19). Some studies suggests that FNA, a simple examination, can detect many metastatic thyroid tumors and identify the origin of some tumors. However, other literature indicates that FNA has a relatively high false-negative rate (up to 28.7%) for secondary thyroid carcinoma (17, 18). If cytomorphological examination fails to confirm the diagnosis, immunohistochemistry, histochemistry, or electron microscopy can be performed. The detection of thyroglobulin and calcitonin (CT) can exclude primary thyroid carcinoma to a certain extent. Nevertheless, it is worth noting that identifying tumors secondary to ccRCC and breast cancer can be challenging, as these tumors’ manifestations are often very similar to the follicular glandular structure composed of clear cells, which is common in thyroid nodules (4, 20).

When thyroid nodules are diagnosed as secondary thyroid cancer, a multidisciplinary team (MDT) should be convened for discussion. Treatment options for metastatic thyroid carcinoma include surgery, chemotherapy, and radiotherapy. The choice of treatment should be based on multiple factors, including the metastatic site, extent of invasion, and surgical risk. Thyroid metastases are a rare phenomenon. Although the overall prognosis is poor, surgical intervention can yield certain positive prognostic benefits. A recent single-center study showed that the median overall survival of the renal cell carcinoma group after surgery for secondary thyroid cancer was 27 months (minimum: 10 - maximum: 44) (21). In this case, the patient had compression symptoms mainly due to the excessively large nodule, and surgery can significantly improve the patient’s current quality of life. There are still controversies in the published literature regarding the therapeutic effect of surgery. Some scholars believe that surgery has no statistically significant impact on survival time, and surgical treatment is often used for palliative care or local lesion control (22). Other scholars argue that surgical treatment may be a means of long-term disease control or even cure when patients have solitary thyroid metastases (4). However, when tumors cause compressive symptoms, active surgical intervention should be performed to improve the patient’s quality of life. For patients with extensive metastases or unresectable tumors due to local invasion, chemotherapy or radiotherapy should be considered. Compared with other metastatic tumors, RCC has limited sensitivity to chemotherapy. Therefore, the current cornerstone of RCC treatment is based on targeted therapy and immunotherapy. Among them, the targeted drug Sunitinib has been proven to be very effective in the management of metastatic RCC (4).

Due to the rarity of metastatic thyroid carcinoma, there are only few case reports in the current literature, and no definitive reference standards exist for its surgical management and therapeutic efficacy. Therefore, it is extremely necessary to conduct a comprehensive assessment of the patient’s clinical symptoms, medical history, and examination results before surgery to develop a personalized diagnosis and treatment plan. At present, further research and the establishment of a consensus on the disease are still needed to develop a standardized diagnosis and treatment methods for RCC with thyroid metastasis. This article reports a case of post-surgically confirmed thyroid metastasis from RCC and summarizes the diagnostic and therapeutic experience for this condition by reviewing the relevant literature (23, 24).

## Conclusion

Metastatic thyroid carcinoma is relatively rare in clinical practice, but RCC is the most common primary site. This case of ccRCC metastasizing to the thyroid gland 12 years after right nephrectomy further highlights that metastatic thyroid lesions from renal cell carcinoma may have a long latent period, and thyroid nodule screening is essential for long-term follow-up of patients with a history of RCC. It is crucial to distinguish between primary and secondary thyroid carcinoma, and clinicians should pay close attention to patients with thyroid nodules who have a history of primary carcinoma. FNA is an important method for differentiating between primary and secondary thyroid carcinoma. Treatment measures should be based on a comprehensive assessment of the individual patient’s condition, and there are still controversies regarding surgical management strategies and efficacy. Targeted therapy has been proven effective.

## Data Availability

The original contributions presented in the study are included in the article/supplementary material. Further inquiries can be directed to the corresponding authors.
